# Genetic commonality of macrolide-resistant group A beta hemolytic streptococcus pharyngeal strains

**DOI:** 10.1186/1476-0711-8-33

**Published:** 2009-12-01

**Authors:** Angela L Myers, Mary Anne Jackson, Rangaraj Selvarangan, Richard V Goering, Christopher Harrison

**Affiliations:** 1 Children's Mercy Hospitals and Clinics, University of Missouri-Kansas City School of Medicine, KC, MO, USA; 2 Creighton University Medical Center School of Medicine, Omaha, NE, USA

## Abstract

**Background:**

Group A beta hemolytic streptococcus (GABHS) pharyngitis is a common childhood illness. Penicillin remains the gold standard therapy, but macrolides are indicated for the penicillin allergic patient, and are often used for convenience.

**Methods:**

We conducted a surveillance study of children with pharyngitis and positive streptococcal rapid antigen testing from 10/05 to 10/06 at 2 sites (A & B). Demographics, treatment, and resistance data was collected and compared to previous data from 2002. Erythromycin (EM) resistance was determined by disk diffusion and E-test on 500 isolates. Pulse field gel electrophoresis (PFGE) was performed to measure genetic relatedness of isolates. StatXact version 8 software (Cytel Inc., Cambridge, MA) was utilized to perform Fisher's exact test and exact confidence interval (CI) analysis.

**Results:**

There were no differences in resistance rates or demographic features, with the exception of race, between sites A & B. EM resistance was 0 in 2002, 3.5% in 2005-06 at site A, and 4.5% in 2005-06 at site B. 3/7 and 3/9 had inducible resistance at A and B respectively. 8 isolates had relatedness ≥80%, 5 of which were 88% homologous on PFGE.

**Conclusion:**

Community macrolide resistance has increased following increased macrolide use. These results may have treatment implications if use continues to be high.

## Background

Group A streptococcal pharyngitis (GABHS) is a common childhood infection that is most frequent in the school age child [1]. Antimicrobial therapy is pursued largely for the prevention of rheumatic fever, and organism eradication virtually eliminates this risk [2]. Penicillin is the gold standard therapy for GABHS infection, and penicillin resistance has never been documented [3]. Macrolides are recommended for the penicillin allergic patient. However, they are being increasingly used for convenience, due to once daily dosing, shorter course of therapy, and perhaps better taste. As macrolide use has increased, resistance has been noted with resultant bacteriologic failures [3-5].

There are two different macrolide resistance mechanisms that Group A streptococcus may carry. The first is an active efflux mechanism caused by the macrolide efflux gene, or mef A gene, which confers resistance to 14 and 15 membered macrolides, but not to 16 membered macrolides, lincosamides, or streptogramin b [3,5]. This is referred to as the M phenotype. The second is caused by erythromycin resistance methylase, or erm gene. The erm gene causes target site modification within the 50s ribosomal subunit. It is expressed in either an inducible or constitutive manner, which results in resistance to macrolides, lincosamides, and streptogramin b antibiotics [6].

Reports of macrolide resistance prevalence are varied from country to country throughout the world. Portugal has reported resistance rates of 27%, Belgium 13%, Spain 30%, and Italy 40% [4,6-8]. While widespread macrolide resistance has been reported in several European countries, low level resistance has been predominantly reported in the US [9]. However, more recent studies have shown an increase in resistance rates to 6-7%, with pockets of higher resistance at differing times between 10-20% [10-12]. This increase in resistance has followed an increase in macrolide use on a national level [13]. Both inpatient and outpatient macrolide prescriptions increased in our institution by 11% and 15% respectively from 2002-04. Our study is an evaluation of local macrolide resistance in 2 separate clinical settings; an urban teaching hospital, and a community pediatric office, with comparison of local previous macrolide resistance data.

## Methods

This was a prospective surveillance study of 400 children with positive rapid streptococcal antigen testing, and symptomatology consistent with streptococcal pharyngitis. A power calculation was performed and revealed that with a sample size of 400 we would have 90% power to detect a 10% shift in macrolide resistance prevalence from near zero to 10%. Institutional review board approval was obtained.

Our urban teaching institution was designated as site A, and the community pediatric office was designated as site B. Convenience sampling of 8 specimens per week was obtained from each site from October 2005 to October 2006. Specimens were inoculated on Trypticase-Soy agar with 5% sheep blood. (Remel Inc., Lenexa, KS) Isolates were confirmed to be GABHS by latex antigen agglutination. Susceptibility testing was performed by double disc (D- test) diffusion method, which allowed for identification of phenotype in resistant isolates [14]. D-testing was performed with a 2 μg clindamycin disc and a15 μg erythromycin disc placed 12 mm apart, with subsequent evaluation for zone blunting around the clindamycin disc. Resistant isolates were then further tested to confirm minimum inhibitory concentration (MIC). Standard Clinical and Laboratory Standards Institute (CLSI) plating and susceptibility techniques were utilized, as well as breakpoints to determine resistance [15,16]. Baseline demographic characteristics including age, race, payor status, treatment, and zip codes were collected. Race was classified by parent report, and was included to characterize the patient population at each site.

Pulse field gel electrophoresis (PFGE) was performed on *in situ Sfi*1 digests of chromosomal DNA from all available resistant isolates using a CHEF DR III System (Bio-Rad, Hercules, CA) at 6 V/cm, 14°C, 120° included angle, with switching from 5 to 15 s for 10 hours, followed by switching from 15 to 60 s for 13 hours [17,18]. *Sfi*1 is a restriction enzyme that produces 5-7 well separated DNA fragments. [19]. Images of ethidium-bromide stained gels were archived using a Bio-Rad Gel Doc 1000 System. PFGE profiles were analyzed using BioNumerics v 4.01 (Applied Maths, St-Martens-Latern, Belgium). Isolates were given specific strain designations based on at least 80% similarity. In addition PFGE was performed on 2 susceptible isolates obtained within 2 weeks of each resistant isolate, in order to make direct genetic comparison of each resistant isolate to current circulating susceptible isolates. Comparison was made between site A and site B, as well as previous site A data from 2002 using Fisher's exact test and exact confidence interval (CI) analysis. We used a two-sided alpha level of 0.05 and 95% confidence limits throughout. These analyses used StatXact version 8 software (Cytel Inc., Cambridge, MA).

## Results

Sites A and B showed no significant differences in demographic patient characteristics on age, sex, payor status, and treatment (Table 1). There was a difference in racial composition with 30% white, 20% Hispanic, 47% African-American, 1% Asian, and 2% Other at Site A, and 80% white, 4% Hispanic, 7% African-American, 0% Asian, and 8% Other at Site B (p = 0.0001).

**Table 1:** Demographic and Treatment Data

	Site A	Site B
Median/Mean age	6 yrs/7.5 yrs	6 yrs/7.5 yrs
Male	104/200 (52%)	105/200 (53%)
Caucasian	61/200 (30%)	161/200 (80%)
Medicaid	131/200 (65%)	108/200 (52%)
Penicillin/amoxicillin	178/200 (91%)	179/200 (90%)
Oral cephalosporin	8/200 (4%)	20/200 (10%)
Other/none	14/200 (7%)	1/200 (0.5%)

There were a total of 16 resistant isolates, 7 from site A in 2005-2006 (3.5%), and 9 from site B in 2005-2006 (4.5%) (Table 2). There was no difference in resistance between the two sites (p = 0.80, 95% CI -0.20, 0.31). No isolates with complete resistance were found at site A in 2002, and the increase in resistance from 2002 to 2005-2006 at site A alone was not statistically significant (p = 0.1, 95% CI -0.11, 0.41). However, the combination of sites A and B revealed a statistically significant increase in resistance (p = 0.05, 95% CI 0.004, 0.25). There were an additional 4 isolates with intermediate resistance noted in 2005-2006, 1 and 3 at site A and B respectively. There were two isolates with intermediate resistance noted in 2002.

**Table 2:** Current GABHS Resistance Rates

	Site A	Site B
Macrolide-Susceptible	192/200 (96%)	188/200 (94%)
Macrolide-Intermediate	1/200 (0.5%)	3/200 (1.5%)
Macrolide-Resistant	7/200 (3.5%)	9/200 (4.5%)
Inducible Clindamycin Resistance^a^	3/7 (42.9%)	3/9 (33.3%)

^a ^Determined by double disk diffusion (D-test)

D-zone testing revealed inducible clindamycin resistance, indicating erm phenotype, in 43% (3/7) of the isolates at site A, and 33% (3/9) at site B. (Table 2) Macrolide only resistance was found in the 10 remaining isolates, which denotes Mef A phenotype. MIC's for erm resistant strains were uniformly >256 μg/ml and 12-32 μg/ml for the Mef A phenotype. Constitutive resistance was not found.

Seasonal differences in the resistance rate were evaluated by noting the number of resistant isolates found per month, and by dividing the year into 4 seasons. The seasons were divided into December, January and February for winter months, March, April, and May for spring months, June, July, and August for summer months, and September, October, and November for fall months. There were a small number of resistant isolates seen in the summer and fall seasons, with only 2 and 3 being noted respectively. However, an increase in the number of resistant isolates was seen in the winter and spring months at 4 and 7 respectively.

**Figure 1:**
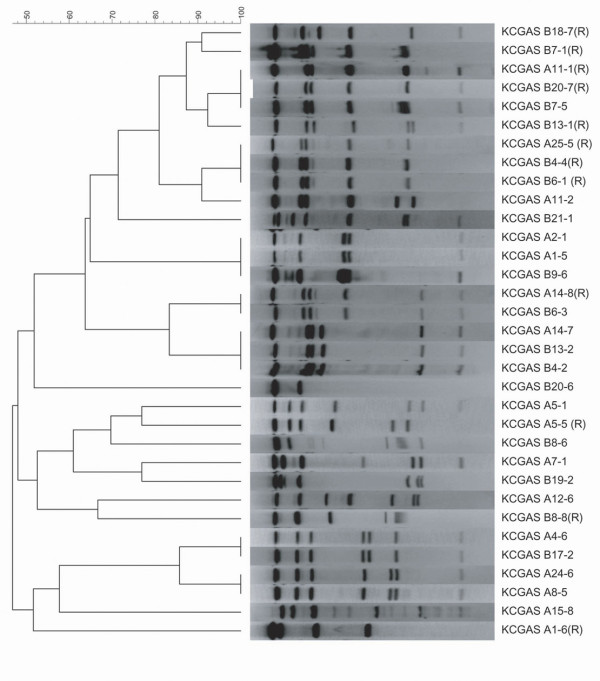
**PFGE of macrolide-resistant and susceptible *Streptococcus pyogenes *isolates**. PFGE was performed on 2 susceptible isolates obtained within 2 weeks of each resistant isolate at the corresponding site for genetic comparison. Each isolate is identified by site of origin (A or B), week in which it was obtained (1-26), and the isolate number within the week (1-8). The dendogram, to the left, denotes the percent of genetic relatedness between resistant and susceptible isolates. The level at which the vertical line transect the horizontal line from the PFGE of each isolate determines its homology based on the percent scale above the dendogram. Twelve resistant and 21 susceptible isolates are pictured. Resistant isolates are identified as (R).

**Figure 2:**
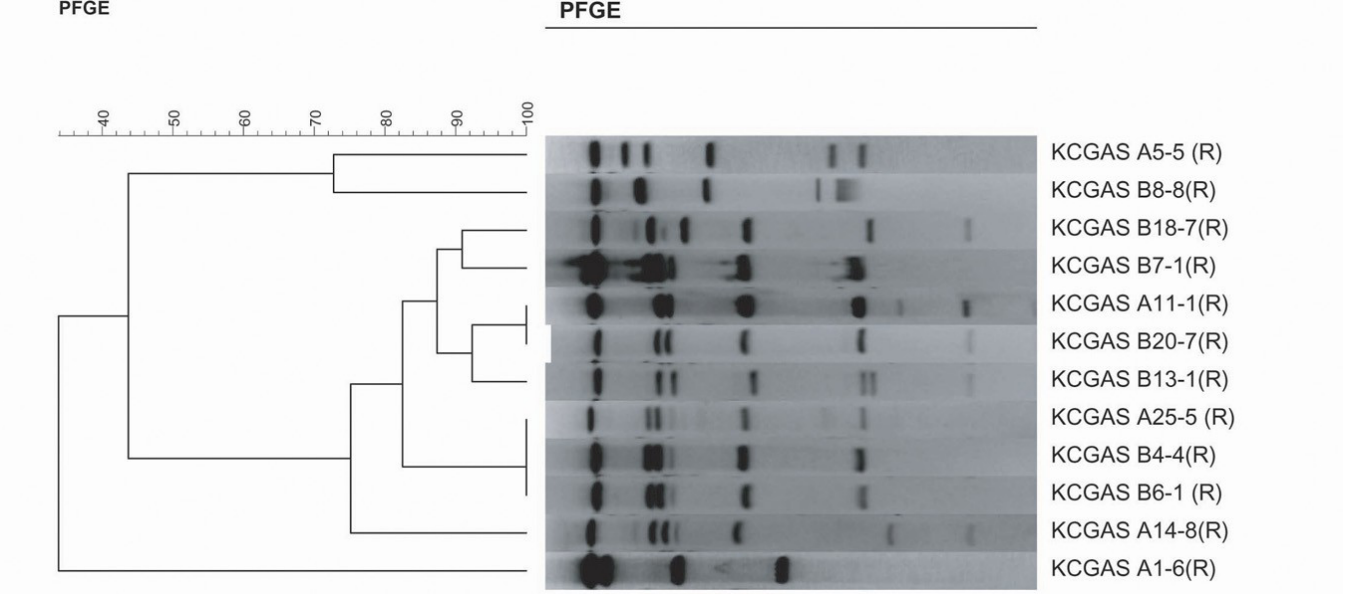
**PFGE of macrolide-resistant *S. pyogenes *isolates**. The dendogram, to the left, compares the percent of genetic relatedness among resistant isolates. The level at which the vertical line transect the horizontal line from the PFGE of each isolate determines its homology based on the percent scale above the dendogram. Twelve resistant isolates are pictured. Each isolate is identified by site of origin (A or B), week in which it was obtained (1-26), and the isolate number within the week (1-8). All isolates are identified as (R) depicting their resistance.

PFGE was initially performed with traditional *Sma*I restriction enzyme, commonly employed for analysis of Gram positive isolates. However, the macrolide resistant strains of the mef A phenotype were not cleaved, necessitating the use of *Sfi*1 enzyme on all isolates for PFGE analysis. [20] Twelve resistant isolates were available for PFGE performance. An additional 21 susceptible isolates underwent PFGE testing for comparison of potential clones. (Figure 1) Isolates were classified by site, week number of the study, and numbered 1-8 from the respective week. Eight of the 12 resistant isolates were found to have ≥80% homology, 6 of which were obtained from site B. (Figure 2) However, one of the patients who had a related strain isolated at site A lives within the site B zip code area. Five of the 8 isolates were in clusters with ≥88% homology, and 7 isolates were D zone test negative demonstrating M phenotype resistance. (Figure 2) We compared PFGE patterns of relatedness among resistance genes in relation to their geographic proximity within the zip code areas surrounding site A and site B. There were no genetically related isolates from site A (0/5), however 6 of the 7 (85%) resistant isolates from site B that underwent PFGE had homology of ≥ 80%.

## Discussion

Macrolide resistance was found in our community, which represents a difference in findings from a previous analysis in 2002. Our rate of macrolide resistance was noted to be similar to rates reported nationally. Local resistance, MIC ≥1 μg/ml, has increased from zero in 2002 to 4.5% in 2005-06, and has become apparent after a documented increase in macrolide use. This increase is not as significant as has been noted in other countries with high rates of macrolide use [4,6-8,21,22]. However, those countries had higher rates of macrolides use than is typically seen in the US, and therefore should be expected to have an elevated resistance rate that correlates with their use. Nevertheless, this data confirms that as rates of antibiotic use increase, resistance rates increase concomitantly. This data then underscores the importance of judicious antimicrobial use.

Nearly 40% of resistant isolates were D-zone test positive, which signifies clindamycin resistance in 1.5% of our GABHS isolates. Routine D-zone testing has not historically been part of GABHS testing, as GABHS is uniformly susceptible to penicillin, and typically requires no further workup past the speciation stage. However, a number of invasive GABHS infections occur yearly with a small percentage of deaths, which makes knowledge of susceptibilities of greater importance. Clindamycin has been utilized in the treatment of invasive infections thought to be caused by a toxin producing strain of GABHS for down regulation of toxin production [23-25]. This makes complete susceptibility testing an important feature when choosing therapy for infection. Although Kirby Bauer testing would provide knowledge of constitutive clindamycin resistance; it would provide no information regarding inducible clindamycin resistance. This information is important, as it may serve to alter the clinical care of the patient in the setting of invasive infection where the length of therapy is typically prolonged, raising the likelihood of inducible resistance resulting in treatment failures during the course of treatment.

Previous literature has reported difficulty in enzymatic degradation of Mef A resistant GAS isolates with *Sma*I enzyme [14]. We encountered the same difficulty, necessitating repeating PFGE testing with *Sfi*I enzyme for all isolates. The inability of *Sma*I to degrade resistant isolates has been theorized to be due to a modification within the genetic element that encodes the M-resistance phenotype [20]. Out of 5 resistant isolates, 4 (≥80%) were found to be D-zone test negative which indicates M phenotype of resistance. Resistance at site A was genetically diverse, but site B isolates were found to have more homology overall, with 6/7 (85.6%) isolates revealing ≥80% similarity, indicating the likelihood of a single clone [19].

Although this was a large sample size, we detected a small number of resistant isolates, which limits the level of statistical precision. Still, our data reveal a trend in our local community that mirrors national resistance rates. Two distinct areas of the community were evaluated which allows for broader generalizations regarding resistance rates overall than would be reasonable if only one site participated in the study.

It was an interesting finding that site B had increased homology of the resistant isolates when compared to site A. This may be related to site B being a relatively smaller community than site A with the potential for easier spread of a single resistant clone. This theory would be better tested with comparison of several smaller community sites along with obtaining an adequate sample size to appropriately power the study.

## Conclusion

Macrolide resistance has emerged in our local community, as well as on a national level. Although the overall percentages are not high at this point, it is becoming an increasingly important problem with continued high levels of macrolide use for upper respiratory tract infections. These results underscore the importance of identification of a bacterial infection prior to antibiotic use, with subsequent susceptibility testing on all invasive isolates.

## Abbreviations

(GABHS): Group A Beta hemolytic Streptococcus; (EES): Erythromycin; (PFGE): Pulse Field Gel Electrophoresis; (MIC): Minimum Inhibitory Concentration; (D- zone test): Double disk diffusion; (CLSI): Clinical Laboratory Standards Institute; (CI): Confidence Interval; (mef): macrolide efflux; (erm): erythromycin resistance methylase.

## Competing interests

The authors declare that they have no competing interests.

## Authors' contributions

ALM developed the study concept, obtained IRB approval and grant funding, specimen collection, maintenance of database, PFGE of isolates, and manuscript preparation. MAJ aided in study design, grant writing, and manuscript preparation. RS helped with study design, carried out E-testing and D- testing in the microbiology lab, and participated in manuscript preparation. RVG supplied lab space and equipment, as well as aiding with carrying out of PFGE testing of isolates and interpretation of results. CJH helped with study design, statistical testing and interpretation, and manuscript preparation. All authors read and approved the final manuscript.
